# Finger drawing on smartphone screens enables early Parkinson’s disease detection through hybrid 1D-CNN and BiGRU deep learning architecture

**DOI:** 10.1371/journal.pone.0327733

**Published:** 2025-07-14

**Authors:** Zhaohui Zhu, E Wu, Pengfei Leng, Jiajun Sun, Mingming Ma, Zhigeng Pan

**Affiliations:** 1 School of Public Administration, Hangzhou Normal University, Hangzhou, China; 2 School of Computer Science and Technology, Huaiyin Normal University, Huaian, China; 3 Rehabilitation and Nursing School, Hangzhou Vocational & Technical College, Hangzhou, China; 4 School of Computer Science and Technology, Zhejiang University of Water Resources and Electric Power, Hangzhou, China; 5 Department of Neurology, Hangzhou Red Cross Hospital, Hangzhou, China; 6 School of Artificial Intelligence, Nanjing University of Information Science and Technology, Nanjing, China; 7 AI Lab, Nanjing MUST Science and Technology Research Institute, Nanjing, China; Politecnico di Torino, ITALY

## Abstract

**Background:**

Parkinson’s disease (PD), a progressive neurodegenerative disorder prevalent in aging populations, manifests clinically through characteristic motor impairments including bradykinesia, rigidity, and resting tremor. Early detection and timely intervention may delay disease progression. Spiral drawing tasks have been established as effective auxiliary diagnostic tools. This study developed a hybrid deep learning model to analyze motion data from finger drawings of spiral and wave lines on smartphone screens, aiming to detect early Parkinson’s disease.

**Methods:**

We recruited 58 age-matched participants (28 early idiopathic PD patients: 68.4 ± 5.7 years; 30 healthy controls: 68.0 ± 4.5 years) for two smartphone-based drawing tasks (spiral and wave). A custom-developed app recorded finger touch coordinates, instantaneous movement speed, and timestamps at a sampling frequency of 60 Hz. Our hybrid model combined multi-scale convolutional feature extraction (using parallel 1D-Convolutional branches) with bidirectional temporal pattern recognition (via gated recurrent unit [GRU] networks) to analyze movement abnormalities and detect the disease.

**Results:**

The proposed model demonstrated robust diagnostic performance, achieving a cross-validation accuracy of 87.93% for spiral drawings (89.64% sensitivity, 86.33% specificity). Wave drawings yielded 87.24% accuracy (86.79% sensitivity, 87.67% specificity). The integration of both tasks achieved 91.20% accuracy (95% CI: 89.2%−93.2%) with balanced sensitivity (91.43%) and specificity (91.00%).

**Conclusion:**

This study establishes the technical feasibility of a hybrid deep learning framework for early PD detection using smartphone-captured finger motion dynamics. The developed model effectively combines one-dimensional convolutional neural networks with bidirectional GRUs to analyze drawing tasks. Distinct from existing approaches that rely on clinical rating scales, neuroimaging modalities, or stylus-based digital assessments, this telemedicine-compatible method requires only bare-finger interactions on consumer-grade smartphones and enables operator-independent assessments. Furthermore, it facilitates cost-effective and convenient PD assessment in remote healthcare and patient monitoring, particularly in resource-limited settings.

## 1. Introduction

Parkinson’s disease (PD), the second most prevalent neurodegenerative disorder globally, currently affects over 10 million individuals with increasing incidence strongly correlated with global population aging trends [1]. This progressive condition stems from dopaminergic neuron degeneration in the substantia nigra pars compacta, leading to striatal dopamine depletion and subsequent manifestations of cardinal motor symptoms (resting tremor, bradykinesia, rigidity, postural instability) alongside non-motor manifestations such as autonomic dysfunction, sleep disturbances, and cognitive decline [2,3]. Early clinical presentations often involve subtle motor abnormalities such as unilateral resting tremors, micrographia, and hypomimia, progressively impairing functional independence and quality of life [3]. Conventional diagnosis has traditionally required extended observational periods (weeks), with studies reporting misdiagnosis rates approaching 25% in typical clinical populations [4]. This diagnostic uncertainty has driven the development of computer-aided systems aimed at accelerating detection timelines to facilitate early therapeutic interventions.

While current neuroimaging modalities such as dopamine transporter SPECT scans demonstrate high diagnostic specificity [5], their clinical utility is constrained by high costs, radiation exposure, and limited availability in resource-limited settings. This limitation has spurred research into quantitative motor biomarkers derived from the digitized assessment of fundamental movements [6–9]. Graphomotor tasks, such as spiral drawing and meander tracing, have proven particularly sensitive for capturing subtle kinematic abnormalities in PD through multi-dimensional analysis of movement dynamics [10–13]. The neurophysiological basis lies in the integration of basal ganglia-cortical circuits required for precise visuomotor coordination, making these tasks effective proxies for detecting dopaminergic dysfunction [14–16].

Existing approaches for quantifying digital hand motion biomarkers fall into three main technical paradigms: 1) Paper-based analysis employing computer vision to quantify deviations in static trajectory [17,18], but limited by the loss of temporal dynamics; 2) Stylus-digitizer systems capturing high-frequency kinematic data (100–1000 Hz) using specialized tablets [10,19,20], achieving diagnostic accuracy up to 90.7% using deep learning architectures [19]; 3) Smartphone-based or IoT-based inertial sensing, quantifying resting tremor through analysis of device motion patterns or tapping tasks [21–25]. Despite their diagnostic potential, these approaches encounter critical limitations: paper-based methods discard crucial temporal information, stylus-based systems require specialized hardware uncommon in clinical practice, and inertial sensing lacks controlled, task-specific motor challenges relevant to Parkinson’s disease.

Recent advances in deep learning offer new opportunities for analyzing complex spatiotemporal patterns in motor tasks [26,27]. Hybrid architectures combining convolutional neural networks (CNNs) with recurrent units (e.g., LSTM, GRU) have shown significant promise in decoding kinematic time series [19,28,29]. Bidirectional gated recurrent units (BiGRUs) enhance temporal modeling by processing sequential data in both forward and reverse directions, effectively capturing both preparatory and compensatory movement phases [29]. However, existing implementations of these hybrid models predominantly rely on stylus-based input devices, limiting their scalability for population screening.

Despite technological advancements, three persistent challenges hinder the widespread clinical adoption of digital motor biomarkers. First, hardware dependency is a critical limitation: specialized devices (e.g., digitizing tablets, IoT sensors) require dedicated clinical infrastructure uncommon in routine practice, while paper-based methods lack the temporal motion recording capabilities essential for detecting early-stage abnormalities. Second, task overspecialization compromises diagnostic validity. For instance, while inertial sensing is effective for quantifying resting tremors, it cannot assess visuomotor coordination deficits captured by controlled drawing tasks. Third, although multi-task approaches theoretically enable complementary feature extraction, current implementations rarely integrate kinematic features derived from multiple distinct motor tasks.

To address these technological limitations and overcome the three critical barriers to clinical translation, we developed a smartphone-based deep learning framework with three innovations: (1) The proposed 1D-CNN-BiGRU hybrid model synergistically combines multi-receptive field feature extraction with bidirectional temporal dependency learning. Parallel convolutional kernels in the 1D-CNN layer capture local kinematic patterns across multiple timescales. The resulting multi-scale features are then processed by the BiGRU layer to model long-range temporal dependencies, effectively balancing the capture of micro-movement details with macro-motion context. A sliding window strategy augments the training data while mitigating overfitting. (2) The synergistic analysis of both spiral and wave drawing tasks captures PD-induced movement abnormalities across distinct motor contexts. Spiral trajectories primarily reveal kinematic instability in fine motor control during sustained motion, whereas wave patterns specifically detect coordination deficits in acceleration-deceleration transitions. This dual-task fusion enhances early-stage detection by quantifying complementary aspects of visuomotor dysfunction that manifest differently across task demands. (3) The framework utilizes finger-drawn trajectory data captured directly via standard smartphone touchscreens. This approach facilitates remote monitoring and enables self-administered assessments, leveraging the ubiquity of consumer smartphones to ensure accessibility without specialized hardware.

The remainder of this paper is structured as follows: Section 2 (Related works) reviews prior studies in depth. Section 3 (Methods and materials) details the experimental protocol, data acquisition system, and model architecture. Section 4 (Experimental results and analysis) presents comparative performance evaluations across multiple task modalities. Sections 5 (Discussion) discuss clinical implications, technical limitations, and future research directions. Finally, Section 6 (Conclusions) provides a summary of the study.

## 2. Related works

Recent methodological advancements in the detection of Parkinson’s disease have diversified analytical approaches for evaluating handwriting and visuomotor tasks. Pereira et al. [17] established a paper-based dataset of graphical tasks (spirals, circles, meanders) from 55 subjects (37 PD, 18 HCs), yielding 373 samples. Their methodology computationally separated freehand trajectories from reference templates to quantify kinematic abnormalities including tremor amplitude, movement regularity, and stroke discontinuity. Comparing three supervised learning models (Naïve Bayes [NB], Optimal Path Forest [OPF], and Support Vector Machine [SVM]) via 10-fold cross-validation, the NB architecture achieved optimal diagnostic performance with an accuracy of 78.9%. Subsequently, the team developed the HandPD dataset [18], expanding the cohort to 74 PD patients and 18 HC participants, generating 736 graphical samples. Despite implementing advanced computer vision techniques for feature enhancement and optimized classifier iterations (NB/SVM/OPF), the recognition rates unexpectedly declined to 67%. This inverse relationship between sample size and accuracy underscores the inherent limitations of static trajectory measurements, as paper-based methods may obscure dynamic symptoms that become apparent in dynamic assessments, such as velocity fluctuations, pressure dynamics, and intra-stroke hesitations.

Parziale et al. [30] implemented Cartesian Genetic Programming (CGP) to evaluate geometric features extracted from digitized drawings using two datasets: PaHaW [12] (37 PD, 38 HCs) and NewHandPD [31] (31 PD, 35 HCs). The experimental design incorporated digital tablet-acquired data, processing multimodal kinematic signals that included pressure dynamics, velocity gradients, acceleration patterns, and spatial displacement metrics between freehand trajectories and reference templates. Comparative analysis of four models (Decision Tree [DT], Random Forest [RF], SVM, CGP) under 10-fold stratified cross-validation revealed a trade-off between diagnostic interpretability and classification performance, with the CGP-based approach achieving mean accuracies of 71.18% on PaHaW and 80.4% on NewHandPD.

While digitized stylus systems offer precision, their limited accessibility has prompted smartphone-based alternatives. Kuosmanen et al. [21] developed the gamified STOP App, quantifying hand tremors via a 10-second ball stabilization task using an accelerometer, gyroscope, and rotation sensors. Analyzing 2,213 game sessions and 1,856 medication logs from eleven subjects, they identified significant kinematic differences via Wilcoxon rank-sum tests and established a tremor severity index correlating with UPDRS scores (Kendall’s τ = 0.537, z = 30.52, p < 0.001). In a follow-up study [22], the team enhanced the STOP App’s functionality by incorporating spiral drawing and square tracing tasks. The expanded protocol generated 84 digital illustrations (24 spirals/squares from PD patients vs. 18 from HCs). Computational analysis of velocity, completion time, line crossing frequency, and radial/angular speed revealed significantly higher HC accuracy (spirals: 32%, squares: 24%; p < 0.05), though velocity differences were non-significant. However, clinical translation was limited by the modest cohort size (n = 14: 8 PD, 6 HCs) and reliance on manual feature engineering.

Addressing these methodological constraints in digital biomarker development, He et al. [32] leveraged a signal encoding framework to transform triaxial inertial sensor data from smartphones into RGB image representations. Their hybrid CNN architecture integrated residual connections (for gradient flow optimization), multi-head attention mechanisms (enhancing feature discriminability), and squeeze-excitation modules (modeling cross-channel dependencies). Utilizing consumer-grade smartphones with embedded accelerometers and gyroscopes, the study acquired kinematic data from 586 participants (119 PD, 467 age-matched HCs). Through 5-fold cross-validation, the proposed model demonstrated clinically meaningful classification performance with an average AUC of 0.883 on resting-state assessments. While this work successfully validated the feasibility of smartphone-based PD detection, its static image transformation paradigm fundamentally limits temporal dependency modeling, failing to capture disease progression dynamics through spatiotemporal feature learning.

With advancements in artificial intelligence, deep learning-based diagnostic models have emerged as powerful alternatives to traditional manual feature extraction. Ramzani et al. [28] synergistically integrated fuzzy inference mechanisms with a bidirectional long short-term memory (BiLSTM) network to model kinematic time series from the spiral drawing tests. Utilizing a digitized pen system with triaxial force sensors, the study quantified fine motor control through high-fidelity recording of pen-tip coordinates, axial pressure, and grip orientation during spiral tracing tasks. The cohort comprised 77 participants (62 PD vs. 15 age-matched HCs), from whom multidimensional feature sets were extracted through joint time-frequency analysis of drawing kinematics. Implementing a Leave-One-Subject-Out (LOSO) cross-validation protocol with participant-level data partitioning (training: 60%, validation: 10%, testing: 30%), the hybrid model achieved exceptional classification accuracies of 97.0%, 98.5%, and 100% across three distinct spiral-drawing paradigms. Notably, the innovative fusion of fuzzy logic with deep temporal modeling overcame conventional limitations in handling kinematic signal uncertainty. However, the clinical applicability is limited by the small HC cohort and the absence of external validation.

Diaz et al. [29] leveraged dynamic handwriting data from two public datasets (PaHaW and NewHandPD) to develop a hybrid deep-learning architecture for PD detection. Their system employed a 1D-CNN to process handwriting sequences, enhancing local feature extraction in time-series data through two convolutional layers. A bidirectional gated recurrent unit (BiGRU) network was subsequently employed to capture temporal dependencies in handwriting patterns, incorporating past and future information to better understand patients’ behavioral characteristics. Methodologically rigorous validation was implemented through dataset-specific protocols: For PaHaW, stratified 10-fold cross-validation ensured population representativeness; whereas for NewHandPD, a hold-out protocol (65% training, 10% validation, 25% testing) was adopted to assess generalizability across data acquisition systems. The model demonstrated state-of-the-art performance with PaHaW: 93.75% accuracy and 93.12% AUC; NewHandPD: 94.44% accuracy and 98.25% AUC on spiral-drawing tasks. Notably, the architecture enhanced by BiGRU demonstrated a significantly better ability to capture multiscale spatiotemporal relationships in dyskinetic handwriting patterns compared to conventional CNN models. Nevertheless, clinical translation remains challenged by dataset heterogeneity and limited NewHandPD sample size (n = 37).

Zhao et al. [33] proposed a spatiotemporal twin neural network to analyze multimodal handwriting anomalies in PD patients on the NewHandPD dataset and PARKINSON_HW dataset(62 PDs, 15 HCs). Employing a digitized smartpen system equipped with triaxial inertial sensors, the protocol quantified kinematic signatures through a high-frequency sampling of writing speed, axial pressure, and trajectory coordinates. Methodologically, the authors implemented a stratified hold-out protocol with randomized 80:20 data partitioning to preserve population representativeness across heterogeneous data sources. Their metric learning framework contrastively paired multimodal handwriting samples (speed, pressure, trajectory) to optimize inter-class discriminability via similarity-based feature alignment. Empirical validation yielded 92.6% accuracy (93.14% F1-score) on NewHandPD image modality and 90.76% accuracy (92.74% F1-score) on signal modality. Cross-dataset evaluation on PARKINSON_HW showed reduced efficacy (77.04% accuracy, 81.92% F1-score), highlighting domain shift challenges. Critical limitations included severe class imbalance (4:1 PD/HC ratio) and restricted ecological validity due to non-universal smartpen data.

## 3. Methods and materials

This section first introduces data acquisition and subsequently presents the proposed model.

### 3.1. Ethical considerations and participant recruitment

This cross-sectional study received ethical approval from the Ethical Committee of Hangzhou Normal University (Approval No.20230001) and Hangzhou Red Cross Hospital (Approval No.2023[102]) and was conducted in accordance with the Declaration of Helsinki guidelines. Between August 9 and October 27, 2023, we recruited 58 right-handed participants (28 early-stage idiopathic PD patients [Hoehn & Yahr [34] stage 1–2.5; mean age 68.4 ± 5.7 years] and 30 age-matched HCs [68.0 ± 4.5 years]) through neurology clinics and community-based recruitment. PD diagnosis followed UK Parkinson’s Disease Society Brain Bank criteria, excluding subjects who met any of the following criteria:

1) Secondary parkinsonism or atypical variants;
2) Visual impairment affecting task performance;
3) Active psychiatric comorbidities;
4) Upper limb musculoskeletal disorders;
5) Hoehn & Yahr stage greater than 2.5;
6) Age greater than 80 or younger than 45.

All participants provided written informed consent and completed dominant hand assessments during the medication ON state. Key subject demographics are summarized in Table 1 (extended data in S1 File).

**Table 1:** Description of the dataset.

Group	Total subjects	Male	Female	Average age (years)	Average Hoehn-Yahr stage (number of subjects in stages 1, 1.5, 2, and 2.5)	Average PD duration (years)
PD	28	10	18	68.43 ± 5.72	1.66 ± 0.50 (7/9/8/4)	3.57 ± 1.99
HC	30	12	18	68.03 ± 4.46	–	–

### 3.2. Experimental protocol

**Fig 1:**
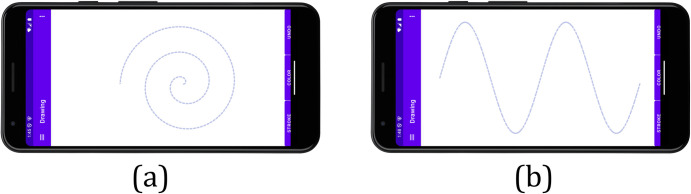
Visual templates displayed during drawing tasks: (a) Archimedes spiral, (b) sinusoidal wave.

Participants performed two visuomotor tasks using a horizontally positioned Vivo X27 smartphone (model: V1829A; screen size: 6.39 inches) with the interface shown in Fig 1. During the drawing tasks, predefined visual templates were displayed on the screen as guiding lines, which participants followed to create their drawings.

**Task 1: Archimedean spiral tracing**

Participants traced the predefined 2.2-inch-diameter spiral guide line (2.5 rotations) from periphery to center, visually following the on-screen template to maintain progressive radial precision (Fig 1a).

**Task 2: Sinusoidal wave tracing**

A displayed sine wave template spanning two periods challenged participants with rapid directional transitions, requiring continuous alignment with the guide line (Fig 1b).

Following a 3-minute practice session to familiarize with guide line tracing, one trial for each task was recorded without speed reminders. A custom Android application captured kinematic time-series data at 60 Hz resolution, including:

1) Touch coordinates (*x*, *y*) in pixel space (1080 × 2340 resolution);
2) Instantaneous velocity components (*v*_*x*_, *v*_*y*_);
3) Timestamps with millisecond precision.

The spiral task showed a mean completion time of 6.68 seconds (variance = 2.69), while the wave task averaged 6.01 seconds (variance = 2.27) (full experimental data in S2 File). Detailed completion times are provided in Table 2.

**Table 2:** Task completion time across study groups.

Task	PD patients (mean±SD, s)	HCs (mean±SD, s)	Combined cohort (mean±SD, s)	Max (s)	Min (s)
Task 1: Spiral	7.77 ± 2.72	6.59 ± 2.67	6.68 ± 2.69	16.63	2.86
Task 2: Wave	6.12 ± 2.20	5.90 ± 2.33	6.01 ± 2.27	11.97	2.80

### 3.3. Data preprocessing pipeline

Raw signals underwent four-stage processing:

1) Kinematic derivation: Calculated linear acceleration (*a *=* Δv/Δt*) and jerk (*j *=* Δa/Δt*).
2) Standardization: Z-score normalization was applied to each feature dimension.
3) Segmentation: Utilized 32-sample sliding windows (0.53 seconds duration) with 75% overlap.
4) Padding: Zero-padding was applied to the final partial segments.

This process resulted in 8-dimensional feature vectors (*x*, *y*, *v*_*x*_, *v*_*y*_, *a*_*x*_, *a*_*y*_, *j*_*x*_, *j*_*y*_) for temporal analysis. To prevent over-optimistic validation, all segments from a subject were confined to a single fold. Splitting occurred at the subject level before segment extraction, ensuring no temporal correlation between training and validation data.

**Fig 2:**
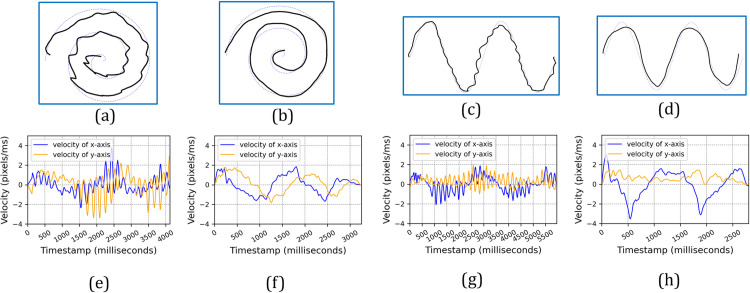
Spiral and wave curves were drawn by a PD patient (a, c) and an HC (b, d), along with their respective velocity profiles. The upper row of plots (a-d) shows the drawing results, while the lower row (e-h) displays the corresponding velocity graphs over time. In the velocity graphs, the blue line represents the velocity of the x-axis, and the orange line represents the velocity of the y-axis.

The comparison of the drawing results and velocity characteristics between a healthy control and a PD patient is illustrated in Fig 2.

### 3.4. Hybrid deep learning architecture

**Fig 3:**
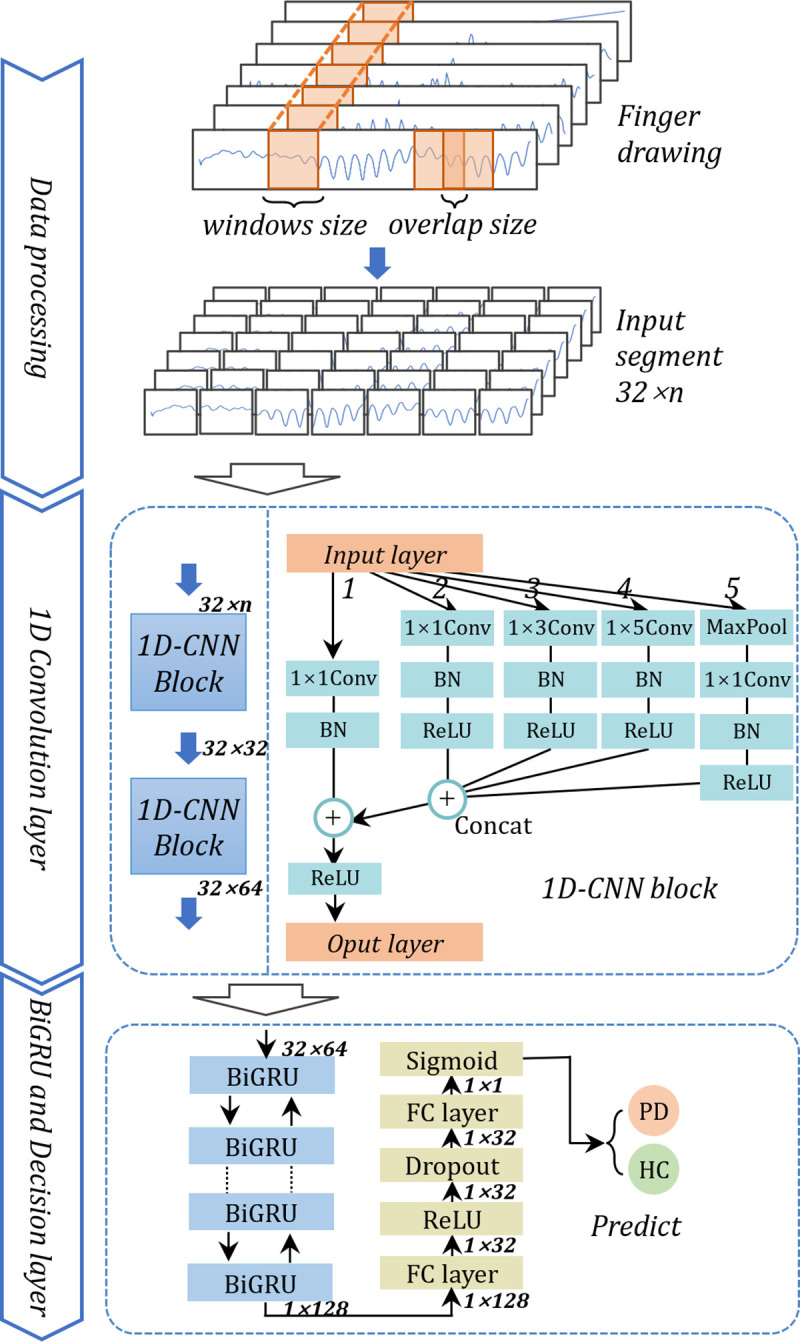
Network architecture of the classification model. The first 1D-CNN block had 4 parallel branches, including 1 × 1, 1 × 3 and 1 × 5 convolutions, along with a 1 × 3 max pooling branch, each with 8 filters used for convolution. The outputs of the four branches were combined with the original input through residual connections. The second 1D-CNN block maintained the same network structure but utilized 16 filters for each branch. The BiGRU layer was configured with 64 hidden units and a dropout rate of 0.3. The decision layer employed a Sigmoid activation function to predict the probability of Parkinson’s Disease.

The proposed hybrid 1D-CNN-BiGRU architecture synergistically integrated multi-scale convolutional feature extraction (via parallel 1D kernels) with bidirectional temporal dependency learning (through BiGRU layers), as illustrated in Fig 3.

#### 3.4.1. Multi-branch 1D-CNN.

The 1D convolutional neural network (1D-CNN) architecture was employed to extract multi-scale temporal features from kinematic sequences. Unlike conventional approaches using fixed receptive fields, our design incorporated parallel convolutions with varying kernel sizes (1, 3, and 5) to effectively capture both immediate micro-oscillations (tremor signatures) and macro-movement patterns (bradykinesia indicators) [19]. This multi-branch configuration enhanced temporal resolution while maintaining parameter efficiency through weight sharing across sequences, a critical advantage for processing long-duration motor recordings (typically lasting 6 seconds per trial) [35]. However, 1D convolution operations may struggle to capture long-distance dependencies due to their limited receptive fields, so combining 1D convolutions with RNNs would be of great benefit.

The first CNN block comprised four parallel branches: three convolutional layers with kernel sizes of 1, 3, and 5, along with a max-pooling branch to enhance feature selection. Each convolutional layer in these branches was configured with 8 filters. The outputs from the four parallel branches were concatenated along the channel dimension and subsequently added to the block input for residual feature fusion [36]. The second CNN block maintained the same network structure but utilized 16 filters in each branch.

#### 3.4.2. Bidirectional GRU.

The recurrent neural network (RNN) processes sequential data through cyclic hidden state transfers. However, RNNs suffer from gradient vanishing and gradient exploding problems when processing long sequences. The long short-term memory (LSTM) network introduced gating mechanisms (input, forget, and output gates) to control the nonlinear transformation of information, thereby alleviating the problem of gradient vanishing. The gated recurrent unit (GRU) simplified these gates into update and reset gates, effectively reducing computational costs and the overall number of parameters.

A bidirectional GRU (BiGRU) consists of two independent GRUs: one for processing the forward sequence and the other for processing the reverse sequence. This architecture captures bidirectional dependencies in the sequence data, avoiding the potential patterns of the preceding or succeeding associations that a single GRU may ignore. The final representation is obtained by concatenating the hidden states of the two GRUs. The processing flow of a single GRU is defined by the following formulas:


$$z_{t}=\sigma (W_{z}\cdot [h_{t-1},x_{t}]+b_{z})$$ (1)

$$r_{t}=\sigma (W_{r}\cdot [h_{t-1},x_{t}]+b_{r})$$ (2)

$${\tilde{h}}_{t}=tanh\left(W_{h}\cdot \left[r_{t}\cdot h_{t-1},x_{t}\right]+b_{h}\right)$$ (3)

$$h_{t}=\left(1-z_{t}\right)\cdot h_{t-1}+z_{t}\cdot {\tilde{h}}_{t}$$ (4)

Where *x*_*t*_ represents the input vector, *z*_*t*_ and *r*_*t*_ represent the update gate and reset gate, respectively, ${\tilde{h}}_{t}$ is the candidate hidden state, *h*_*t*_ represents the hidden state, *W* and *b* represent the weight matrix and bias, respectively, and *σ* represents a nonlinear activation function. BiGRU combines two GRUs and provides a comprehensive evaluation result through the forward and backward predictions of the two GRUs. Its implementation is as follows:


$${\left(h_{i}^{t}\right)}_{forward}=GRU_{forward}\left(s_{i}^{t},h_{i}^{t-1}\right),t\in [1,N]$$ (5)

$${\left(h_{i}^{t}\right)}_{backward}=GRU_{backward}\left(s_{i}^{t},h_{i}^{t-1}\right),t\in [N,1]$$ (6)

$$h_{i}^{t}=\left[{\left(h_{i}^{t}\right)}_{forward};{\left(h_{i}^{t}\right)}_{backward}\right]$$ (7)

To enhance the perception of global features in kinematic time-series data, we utilized a bidirectional GRU with 64 hidden units in each direction to process the output of the 1D-CNN blocks.

#### 3.4.3. Decision layer.

The output of the BiGRU was flattened and then input into a fully connected network (FC), and a sigmoid classifier was used to predict the probability of being diagnosed with Parkinson’s Disease. In order to prevent overfitting during training, a dropout layer was added to the FC layers, and a dropout rate of 0.3 was applied.

### 3.5. Implementation details

The model was implemented using PyTorch 2.4 and Scikit-learn 1.4.2 libraries. An Adam optimizer was used for training, with the learning rate set to 0.001 and a batch size of 4. The workstation utilized for model training and validation was equipped with an Intel® Core™ i9-11900K CPU, 32 GB of RAM, and an NVIDIA GeForce RTX 3090 GPU with 24 GB of VRAM. The complete source code is available in S3 File.

### 3.6. Validation strategy

We empirically adopted a stratified 10-fold cross-validation method to ensure robustness and took the average of all segment predictions in a drawing sample as the final prediction value. A value greater than 0.5 was determined as PD, and the average probability of the spiral and wave models was taken as the subject’s final judgment on task fusion.

## 4. Experimental results and analysis

All experiments employed stratified 10-fold cross-validation with 10 repetitions to ensure statistical robustness. Performance metrics included accuracy (ACC), sensitivity (SEN), specificity (SPE), and F1-score (F1), reported as mean values across validation runs.

### 4.1. Feature engineering analysis

Comprehensive feature ablation studies revealed the utility of biomarkers that depend on the task employed (Table 3). For spiral tracing, velocity-acceleration pairing achieved peak accuracy (87.93%), whereas coordinate-velocity combinations optimized performance on the wave task (87.24%). Jerk features showed limited standalone predictive value (spiral: 74.66%; wave: 79.66%).

**Table 3:** Classification performance of feature combinations across motor tasks.

	Spiral				Wave			
Feature combination	ACC(%)	SEN(%)	SPE(%)	F1(%)	ACC(%)	SEN(%)	SPE(%)	F1(%)
(a) coordinate + velocity	85.86 ± 2.28	80.00 ± 3.97	91.33 ± 2.20	84.49 ± 2.69	**87.24 ± 2.06**	86.79 ± 3.21	87.67 ± 3.95	**86.80 ± 2.09**
(b) all features	86.72 ± 2.99	85.36 ± 4.64	88.00 ± 3.05	86.09 ± 3.27	83.62 ± 1.76	82.86 ± 7.28	84.33 ± 5.78	82.89 ± 2.55
(c) velocity + acceleration + jerk	86.21 ± 3.27	83.21 ± 4.80	89.00 ± 4.72	85.34 ± 3.49	83.27 ± 2.32	83.21 ± 3.21	83.33 ± 3.65	82.77 ± 2.34
(d) acceleration + jerk	74.66 ± 4.88	76.43 ± 5.34	73.00 ± 8.75	74.50 ± 4.39	79.66 ± 3.60	83.93 ± 4.30	75.67 ± 5.58	79.95 ± 3.40
(e) velocity + acceleration	**87.93 ± 3.77**	89.64 ± 5.16	86.33 ± 3.48	**87.73 ± 3.98**	84.31 ± 3.31	82.50 ± 5.40	86.00 ± 2.00	83.47 ± 3.78
(f) coordinate + velocity + acceleration	86.21 ± 2.18	83.93 ± 4.30	88.33 ± 3.72	85.43 ± 2.40	84.14 ± 3.06	86.07 ± 4.05	82.33 ± 4.22	83.97 ± 3.01

### 4.2. Model performance comparisons

For the Spiral classification task, we employed velocity and acceleration feature sets, while the Wave task utilized coordinate-velocity features. Our comparative analysis systematically evaluated performance differences among BiRNN, BiLSTM, and BiGRU architectures with/without 1D-CNN integration under a standardized evaluation framework (Table 4). All models were implemented with identical hyperparameter configurations to isolate architectural effects.

**Table 4:** Model performance across architectures.

	Spiral				Wave			
Model	ACC(%)	SEN(%)	SPE(%)	F1(%)	ACC(%)	SEN(%)	SPE(%)	F1(%)
BiRNN	66.04 ± 4.93	47.50 ± 5.54	83.33 ± 8.94	57.46 ± 5.25	66.04 ± 4.69	57.86 ± 10.2	73.67 ± 7.06	61.73 ± 7.64
BiLSTM	81.72 ± 3.63	83.93 ± 5.59	79.67 ± 5.26	81.57 ± 3.65	83.10 ± 2.41	81.43 ± 7.10	84.67 ± 6.18	82.23 ± 2.83
BiGRU	82.24 ± 3.45	81.43 ± 3.11	83.00 ± 5.46	81.62 ± 3.29	81.55 ± 4.01	82.50 ± 8.51	80.67 ± 4.16	81.01 ± 4.86
1D-CNN + BiRNN	82.76 ± 4.62	81.78 ± 4.05	83.67 ± 6.40	82.14 ± 4.44	75.35 ± 5.56	80.36 ± 6.02	70.67 ± 9.40	75.95 ± 4.94
1D-CNN + BiLSTM	84.83 ± 2.53	83.21 ± 4.80	86.33 ± 2.77	84.06 ± 2.93	85.00 ± 3.08	86.43 ± 5.93	83.67 ± 3.78	84.70 ± 3.46
1D-CNN + BiGRU	**87.93 ± 3.77**	89.64 ± 5.16	86.33 ± 3.48	**87.73 ± 3.98**	**87.24 ± 2.06**	86.79 ± 3.21	87.67 ± 3.95	**86.80 ± 2.09**

The experimental results demonstrated that models integrating 1D-CNN with bidirectional gated architectures (BiGRU/BiLSTM) achieved optimal performance on both Spiral and Wave datasets. The 1D-CNN + BiGRU configuration attained peak accuracy (87.93% ± 3.77) and F1-score (87.73% ± 3.98) on Spiral, while maintaining dominant accuracy (87.24% ± 2.06) on Wave. In contrast, the bidirectional RNN (BiRNN) exhibited the weakest metrics across evaluations. Notably, the integration of 1D-CNN consistently enhanced the performance of BiLSTM/BiGRU, whereas its effectiveness on BiRNN was limited to Spiral tasks, indicating architectural compatibility constraints in extracting complex temporal patterns.

### 4.3. Multimodal fusion performance

**Fig 4:**
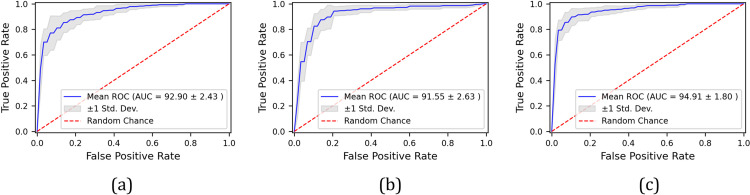
ROC performance of task-optimized feature sets: (a) spiral, (b) wave, (c) fused predictions across 10 runs.

An integrated analysis of the dual-task data yielded superior diagnostic accuracy (91.20%, p = 0.027, 95% CI: 89.2–93.2%) compared to the individual tasks (Fig 4). The fused model demonstrated balanced sensitivity (91.43% ± 3.27) and specificity (91.00% ± 4.72), with an AUC of 0.949 ± 0.018.

### 4.4. Ablation studies

**Fig 5:**
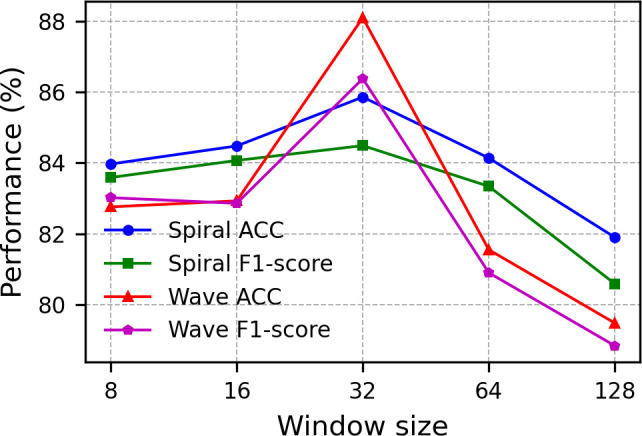
Performance comparison of different window sizes.

**Temporal window size:** The sliding window technique can augment the number of trainable samples when the original dataset is limited in size [19,29]. We evaluated model performance across five representative window lengths, ranging from 8 to 128 time steps, as illustrated in Fig 5. A comparative analysis of the two drawing tasks revealed a consistent pattern: both recognition accuracy and F1 scores initially improved but declined with increasing window size, with the Wave drawing task exhibiting greater performance fluctuations. At smaller window sizes, both tasks exhibited limitations in feature extraction. Peak performance was observed with a 32-time-step window size; beyond this point, continuous deterioration in performance indicated a potential feature dissolution effect associated with larger windows. These observations highlight the necessity for task-specific adaptation of window sizes to accommodate variations in the temporal structure of motion patterns across different drawing activities.

**Fig 6:**
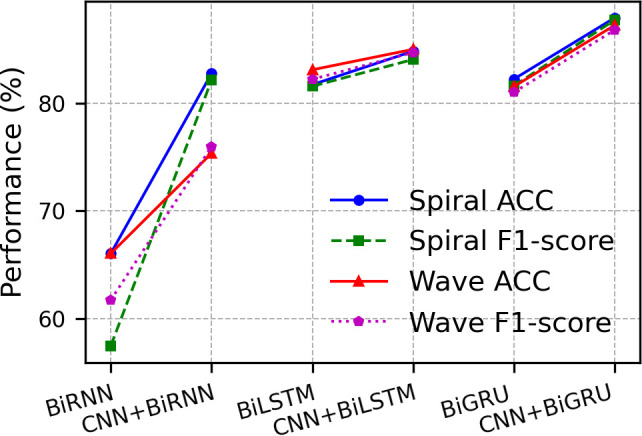
Comparison of model performance with and without convolutional front-end.

**Model architecture:** Since the length of the segment data sequence generated on each drawing sample was not fixed, the BiGRU network that can process indefinite long-time series data was the core of the entire model. We first compared the performance of BiLSTM, BiGRU, and BiRNN in directly processing raw kinematic time-series data. From the performance indicators in Table 4, the BiGRU module had better accuracy performance. On this basis, we compared the performance of BiLSTM, BiGRU, and BiRNN after adding the same 1D-CNN modules as the network front end (Fig 6). From the results, 1D convolution can significantly improve the capabilities of the recurrent network model.

The integration of a single-layer CNN module with the BiGRU architecture was observed to enhance classification accuracy by 2.2 percentage points. Further expanding the model to a dual-layer CNN configuration yielded an incremental improvement of 1.2 percentage points. However, a three-layer CNN architecture exhibited a performance degradation of 1.7 percentage points compared to the dual-layer counterpart. Consequently, the optimal architecture consisted of two layers, balancing model complexity and predictive capability.

**1D-CNN Ablation:** To investigate the contribution of individual branches in the 1D convolutional neural network (1D-CNN) to classification performance, we conducted a systematic ablation study. The original network, comprising two sequential 1D-CNN layers, was modified to establish a baseline structure with a single 1D-CNN layer, thereby isolating the effects of hierarchical feature learning. Each subsequent ablation variant was derived by sequentially removing one data-processing branch while retaining the remaining architecture (see Fig 3 for branch indexing). Models without specific components are denoted as ‘w/o’ (e.g., w/o Branch 1). All experiments utilized the original dataset and identical 10-fold cross-validation protocols to ensure comparability. Performance metrics, including accuracy (ACC), sensitivity (SEN), specificity (SPE), and F1-score (F1), were evaluated to quantify the impact of each branch on model efficacy (Table 5)

**Table 5:** Ablation study of 1D-CNN architectural components.

	Spiral				Wave			
Model Configuration	ACC(%)	SEN(%)	SPE(%)	F1(%)	ACC(%)	SEN(%)	SPE(%)	F1(%)
Baseline model	84.48 ± 4.43	82.85 ± 8.11	86.00 ± 4.16	83.59 ± 5.21	85.68 ± 3.27	83.92 ± 6.02	87.33 ± 3.26	84.90 ± 3.83
w/o Branch 1	84.13 ± 2.28	83.56 ± 3.27	84.66 ± 3.05	83.56 ± 2.41	82.75 ± 3.53	78.92 ± 4.90	86.33 ± 3.78	81.51 ± 3.95
w/o Branch 2	84.13 ± 1.50	84.28 ± 3.64	84.00 ± 4.16	83.68 ± 1.50	85.00 ± 4.29	85.71 ± 5.05	84.33 ± 5.58	84.66 ± 4.39
w/o Branch 3	82.93 ± 1.96	81.78 ± 4.05	84.00 ± 2.49	82.18 ± 2.26	82.41 ± 3.68	82.85 ± 4.16	82.00 ± 5.41	81.99 ± 3.60
w/o Branch 4	82.41 ± 3.91	82.85 ± 4.16	82.00 ± 4.98	81.99 ± 3.86	83.27 ± 1.89	83.56 ± 3.64	82.99 ± 4.06	82.82 ± 1.88
w/o Branch 5	82.65 ± 2.12	82.19 ± 4.64	83.07 ± 3.76	81.87 ± 2.73	81.89 ± 3.47	82.50 ± 6.47	81.33 ± 5.41	81.41 ± 3.86

As shown in Table 5, the ablation study revealed the functional roles of distinct architectural components in the 1D-CNN, where Branch 1 (residual connection) and Branch 2–5 (multi-scale convolutional/pooling operations) collaboratively enabled robust feature learning. Removing Branch 1 (residual skip connection) significantly degraded waveform classification performance (ACC: 82.75% vs. baseline 85.68%, Δ = 2.93%, p < 0.05; F1: 81.51% vs. 84.90%, Δ = 3.39%, p < 0.05), as its absence disrupted gradient flow during backpropagation, which was particularly critical for maintaining temporal coherence in waveform tasks. However, its minimal impact on spiral classification (ACC: 84.13% vs. 84.48%, Δ = 0.35%, p > 0.05) suggested that spatiotemporal patterns in spiral trajectories relied less on residual shortcuts, likely due to their inherent geometric regularity.

Branch 2 (1D convolution, kernel size = 1) showed negligible performance loss when it was removed (wave ACC: 85.00% vs. 85.68%, Δ = 0.68%, p > 0.05; spiral ACC: 84.13% vs. 84.48%, Δ = 0.35%, p > 0.05), indicating its role in local feature smoothing was compensated by subsequent branches. In contrast, Branch 3 (3-kernel convolution) and Branch 4 (5-kernel convolution) exhibited complementary scale-specific effects. Removing Branch 3 reduced spiral specificity (SPE: 84.00% vs. 86.00%, Δ = 2%, p < 0.05), while the ablation of Branch 4 impaired wave sensitivity (SEN: 82.50% vs. 83.92%, Δ = 1.42%, p < 0.05). The ablation of Branch 5 (max pooling) caused the most consistent declines (ΔF1 = 1.72–3.49%) across tasks (spiral F1: 81.87%; wave F1: 81.41%), demonstrating its critical role in integrating local salient features.

The residual fusion of Branches 1–5 ensured that both high-frequency details (via convolutions) and holistic motion trends (via pooling) were preserved, which was essential for distinguishing subtle PD-related kinematic anomalies. These results validated the necessity of integrating multi-scale convolutions, hierarchical pooling, and residual learning to balance local and global feature interdependencies in motor biomarker extraction.

### 4.5. External validation on NewHandPD

To validate the generalizability of the model on an independent dataset, we conducted external validation using spiral-drawing data from the publicly available NewHandPD [31] dataset. This dataset comprised 35 healthy controls (aged 44.05 ± 14.88 years) and 31 PD patients (aged 57.83 ± 7.85 years), all of whom performed six drawing tasks using a smart pen. In the spiral drawing task, the template in NewHandPD aligned with the 2.5-turn Archimedean spiral design adopted in our study, differing only in drawing scale. The NewHandPD dataset contained six-channel sensor signals, but to ensure feature space consistency with our dataset, we exclusively extracted x- and y-axes acceleration data. The constructed four-dimensional feature vector integrated x/y-axes accelerations with their corresponding first-order temporal derivatives (x/y-axes jerks), maintaining consistency with the feature extraction framework established in our dataset.

Given the high sampling rate of NewHandPD (1000 Hz), we downsampled the data to approximately 60 Hz to match the acquisition conditions of our recordings. Each participant’s four spiral drawing trials were independently segmented into four test samples (sp1, sp2, sp3, and sp4) to ensure sample sizes comparable with our study.

The drawing time statistics for subjects in the NewHandPD dataset were as follows: The average time for the HC group was 11.85 ± 4.36 seconds, while the PD group averaged 24.44 ± 11.88 seconds. Overall, the average drawing time was 17.77 ± 10.76 seconds, with a maximum of 75.5 seconds and a minimum of 3.2 seconds.

Throughout testing, the model architecture and hyperparameters remained unchanged, with no parameter tuning or retraining performed on the external data. Preprocessing steps, including normalization, were rigorously synchronized with those applied to our dataset. Performance metrics, accuracy (ACC), sensitivity (SEN), specificity (SPE), and F1-score (F1), were computed to compare model robustness between datasets, with results tabulated to emphasize the model’s stability across variation in drawing scale (Table 6).

**Table 6:** Cross-dataset performance comparison.

	Spiral Drawing (acceleration + jerk)			
Dataset	ACC(%)	SEN(%)	SPE(%)	F1(%)
Our dataset	74.66 ± 4.88	76.43 ± 5.34	73.00 ± 8.75	74.50 ± 4.39
sp1 (NewHandPD)	78.18 ± 3.46	85.16 ± 3.87	72.00 ± 6.85	78.62 ± 2.81
sp2 (NewHandPD)	78.02 ± 3.84	87.42 ± 3.93	69.71 ± 9.91	79.03 ± 2.55
sp3 (NewHandPD)	74.39 ± 3.67	78.06 ± 4.02	71.14 ± 6.69	74.16 ± 3.26
sp4 (NewHandPD)	76.96 ± 3.44	84.51 ± 4.02	70.28 ± 4.98	77.52 ± 3.30
Avg (NewHandPD)	76.89 ± 3.60	83.79 ± 3.96	70.78 ± 7.11	77.33 ± 2.98

The model achieved a mean classification accuracy of 76.89% on the NewHandPD dataset, marginally outperforming the baseline model (74.66%, Δ = 2.23%, p > 0.05,), thereby demonstrating its generalizability across heterogeneous datasets. The sensitivity and specificity reached 83.79% and 70.78%, respectively, indicating robust performance in identifying Parkinson’s disease patients while maintaining moderate specificity for distinguishing healthy controls.

Despite the promising results, several limitations warrant consideration. First, the difference in spiral scale between NewHandPD and our designed template could introduce variations in acceleration amplitude ranges, although normalization techniques partially mitigated this bias. Second, sensor heterogeneity, such as differences in noise levels and sampling rates across devices, might affect signal fidelity, necessitating further validation of hardware-agnostic generalization. Third, the limited sample size of the external cohort (66 subjects) restricts statistical power, and future studies should prioritize validation on larger, multicenter datasets to enhance reliability.

Despite these challenges, the model demonstrated stable classification performance in cross-dataset scenarios, confirming the feasibility of spiral-drawing kinematic features as biomarkers for PD assessment. These findings provide a foundational technical framework for future clinical deployment.

## 5. Discussion

Parkinson’s disease is a prevalent neurodegenerative disorder characterized by its significant impact on motor control in the central nervous system. The substantia nigra in the midbrain, responsible for dopamine production, plays a critical role in motor planning and execution through its interaction with the striatum. Neuronal degeneration in this region leads to reduced dopamine levels, resulting in striatal dysfunction and subsequent motor symptoms such as tremors, rigidity, and bradykinesia, which impair daily manual activities. Numerous existing studies have utilized writing or drawing tasks to detect PD-related motor abnormalities.

This study developed a deep learning framework based on a hybrid 1D-CNN-BiGRU architecture to differentiate early-stage PD patients from healthy controls using finger motion data captured during smartphone-based drawing tasks. The model extracted multi-scale temporal features through parallel 1D convolutional networks with varying receptive fields, followed by bidirectional temporal modeling via BiGRU. By fusing data from spiral and wave drawing tasks, the framework achieved a classification accuracy of 91.20%, approaching the 94.44% accuracy reported for specialized stylus-based systems [29]. Notably, our approach eliminates the need for dedicated hardware, enhancing scalability for clinical applications. The multi-scale temporal processing compensates for the lower sampling rate of smartphone sensors (60 Hz vs. 200 Hz in digitizer systems [19]), enabling the detection of PD-specific motor patterns such as velocity fluctuations linked to tremors and bradykinesia. This finding demonstrates that consumer-grade devices paired with advanced computational models can achieve clinically comparable sensitivity for the early detection of PD.

Comparisons with stylus-based studies (Table 7) reveal that our system has not yet matched the highest reported performance. This discrepancy may arise from the inherent advantages of stylus use, including superior operational precision, user familiarity, and alignment accuracy with guide lines. In contrast, while finger-based drawing on smartphones is convenient and accessible, it is more prone to limitations such as restricted screen space, finger occlusion, and varying user proficiency. To evaluate cross-dataset generalizability, we performed external validation by aligning feature spaces between datasets, specifically using shared kinematic parameters (x/y-axis accelerations and jerks) from spiral drawings in NewHandPD, and downsampling its raw 1000 Hz sensor data to 60 Hz via our preprocessing pipeline to match the sampling rate of our proprietary dataset. Notably, the model achieved higher performance on the NewHandPD dataset than on our original dataset, highlighting the significant impact that hardware specifications and data acquisition protocols have on model evaluation.

**Table 7:** Comparison of research based on hand kinematic features.

Study	Features	Models	Dataset	Accuracy (%)	Year
Gupta et al. [37]	Kinematic (e.g., velocity, acceleration, jerk), entropic(e.g., Shannon entropy), and energetic	SVM	PaHaW	75.76	2020
Parziale et al. [30]	Temporal (e.g., duration), kinematic (e.g., velocity, acceleration, jerk), dynamic	CGP	PaHaW	71.18	2021
Xu et al. [20]	Stylus signals (e.g., pressure, acceleration in three directions)	Ensemble of RF	NewHandPD	89.4	2020
Diaz et al. [29]	Kinematic (e.g., velocity, acceleration, jerk)	1DCNN+BiGRU	PaHaW	94.44	2021
Zhao et al. [33]	Smartpen signals (e.g., pressure, acceleration in three directions),	ST-siamNN	NewHandPD, PARKINSON_HW	95.86	2023
Ramzani et al. [28]	Smartpen signals (e.g., pressure, acceleration in three directions), Geometric features	BiLSTM+ Fuzzy Inferential Classifier	Proprietary dataset	SST: 97, DST: 98.5, SCTP: 100	2023
Wang et al. [19]	Smartpen signals (e.g., pressure, acceleration in three directions)	LSTM-CNN	DraWritePD	96.2	2024
Proposed Method	Kinematic (e.g., velocity, acceleration, jerk) from the smartphone	1DCNN+BiGRU	Proprietary dataset	Spiral: 87.93, Wave: 87.24, Overall: 91.20	–

Inevitably, variations in drawing speed and style occurred across participants during data collection. To mitigate confounding factors, we implemented template guidance, standardized protocols, as well as pre-task training. Pre-training ensured that PD patients could perform tasks proficiently, reducing artifacts from task unfamiliarity (e.g., hesitation or errors) and enhancing the capture of disease-specific motor abnormalities.

While smartphone-based assessments face inherent hardware heterogeneity (e.g., screen sensitivity, sampling rate variations), our preprocessing pipeline normalized motion data across dimensions, minimizing inter-device and inter-user spatial discrepancies. This shifted the model focus toward PD-specific pathophysiological patterns rather than absolute signal magnitudes, improving cross-device generalizability. Adaptive calibration algorithms are under development to further mitigate hardware-induced variability.

Our approach complements emerging smartphone-based PD detection methods. Alternatives using tapping tests [38,39], voice analysis [40–42], or accelerometer protocols [43–45] have achieved notable performance (reported accuracies: 82–100%). These methods often excel in capturing specific motor or vocal manifestations but may require specialized hardware setups (e.g., fixed phone placement), controlled vocalizations, as well as constrained interfaces. In contrast, our finger-drawing paradigm leverages intuitive, template-guided interactions on unmodified consumer devices, capturing integrated kinematic deficits (tremor, bradykinesia, rigidity) during dynamic visuomotor tasks. While our accuracy (91.20%) is competitive with these modalities, its clinical value lies in ecological validity and task flexibility, requiring only standard touchscreens and mimicking everyday digital interactions. Future multimodal systems could synergistically combine these complementary approaches (e.g., drawing + voice) to enhance sensitivity.

Importantly, our dual-task design provides potential mechanistic insights into the pathophysiology of Parkinson’s disease. The spiral task, characterized by sustained velocity control, revealed motor instability that could reflect impaired dopaminergic modulation of the nigrostriatal pathway, possibly contributing to compromised continuous motor execution [46]. Conversely, the wave task with alternating acceleration-deceleration demands detected phase coordination deficits, which may suggest dysfunctional sensorimotor integration within the cortico-basal ganglia loop [47,48]. While spatial localization of these motor impairments to specific brain regions remains to be elucidated, the synergistic effect of the two tasks not only improves detection sensitivity but also provides preliminary support for investigating distributed dysfunction in the cortico-subcortical networks underlying Parkinson’s disease pathophysiology.

### Current limitations and future directions

Despite progress, several limitations warrant consideration. First, the limited training dataset raises the risks of overfitting to spurious patterns or sensor noise, potentially compromising generalization. To mitigate this, we employed a lightweight BiGRU architecture (64 units) with dropout (rate = 0.3) and L2 regularization, and additional dropout on dense layers. Furthermore, our subject-independent cross-validation mitigates data leakage, but it may reduce the effective training data size. Future studies should prioritize collaborations across multiple centers to expand datasets and explore synthetic data augmentation strategies to improve diversity. While the single-trial design aligns with pragmatic clinical assessments, it may not fully capture intra-subject motor fluctuations inherent to PD. The sliding window approach augments sample size but reflects temporal variability within a single task execution rather than true test-retest reliability. Future studies should integrate longitudinal multi-session designs to disentangle disease progression from transient symptom variability.

Second, fundamental limitations of smartphone sensors compared to the dedicated hardware persist. Variable touchscreen sampling rates (typically 60–240 Hz vs. 200–1000 Hz in high-end stylus systems) may impair temporal feature extraction (e.g., velocity estimation). Temporal misalignment between sensor data and user-perceived interactions, along with screen resolution-induced coordinate quantization errors, could affect spatial feature accuracy. Although temporal normalization and device-specific coordinate scaling were applied, residual hardware-related noise likely contributes to the performance gap between our system (91.20%) and state-of-the-art stylus-based models (94.44–100% [28,29,33]).

Third, while our method demonstrated robustness on a single Android device (Vivo X27), its generalizability to heterogeneous platforms (e.g., iOS vs. Android) and devices with varying sampling rates (e.g., 60–240 Hz) requires further validation. Future work will focus on developing adaptive resampling algorithms to harmonize multi-rate data streams and establishing standardized protocols for cross-platform deployment.

Clinically, the current model is not capable of differentiating between tremor-dominant and akinetic-rigid PD subtypes. While phenotype-inclusive training ensures broad applicability, subtype identification is crucial for personalized treatment. Future efforts will integrate phenotype-specific kinematic thresholds or multimodal data fusion. To address hardware constraints, adaptive resampling algorithms and federated learning frameworks [49] are under development to enhance cross-device robustness.

Expanding datasets through multicenter studies and synthetic augmentation, coupled with passive monitoring of natural touchscreen interactions and ergonomically optimized templates, may further improve diagnostic sensitivity. These steps will facilitate the transition of the framework from controlled validation to real-world clinical deployment, thus bridging the performance gap with dedicated hardware-dependent systems.

## 6. Conclusions

This study established the technical feasibility of a hybrid 1D-CNN-BiGRU deep learning model that utilized smartphone-acquired finger-drawn kinematics as a viable digital biomarker for the early detection of Parkinson’s disease. The convenience and cost-effectiveness of smartphone-based drawing tasks position this approach for scalable implementation in resource-limited settings, thereby enhancing accessibility for early-stage PD screening. Moreover, the findings support the potential integration of this method into telemedicine and home monitoring applications, allowing for continuous assessment of patients in their environments. Our research not only contributes to the growing body of evidence supporting digital biomarkers but also underscores the applicability of advanced computational models in routine clinical practice. Despite certain limitations, such as a relatively small dataset and the need for cross-device validation, the adaptability of our method suggests promising avenues for future research. Continued exploration of this approach can lead to improved diagnostic sensitivity and broader applicability in community settings.

## Supporting information

S1 File Subject demographic information. (XLSX)

S2 File Dataset. (ZIP)

S3 File Python source code. (TXT)
