# Supplementary material for: Finger drawing on smartphone screens enables early Parkinson’s disease detection through hybrid 1D-CNN and BiGRU deep learning architecture
Source: PLoS One. 2025 Jul 14;20(7):e0327733. doi: 10.1371/journal.pone.0327733 (PMC12258557; doi:10.1371/journal.pone.0327733)
Supplement: S2 File — (ZIP) [file pone.0327733.s002.zip › Dataset/data description.docx]

***Subject-Drawing-Data.zip* compression package description**

A total of 58 subjects participated in the test, of which the first 28 were PD patients (numbered 1 to 28) and the last 30 were healthy subjects (numbered 29 to 58).

The compressed file contains two folders, ***Spiral*** and ***Wavy***, each of which contains two subfolders, ***healthy*** and ***patient***, which store the kinematic time-series data files generated by the subjects. The first three digits of the file name are the subject number, followed by the subject type, drawing type, and drawing hand. Each line of data in the file is the screen coordinate value, timestamp, speed of the current touch point, and the acceleration and jerk value generated during the later data processing, that is, (*Coordinate-x, Coordinate-y, timestamp, Velocity-x, Velocity-y, Acceleration-x, Acceleration-y, Jerk-x, Jerk-y*). The timestamp starts from 0 and is in milliseconds. The coordinate value is the position value of the mobile phone screen. If you need to convert it into a standard unit value, you can use the conversion relationship: x_dpi=403.411, y_dpi=401.594.

The demographic information of each subject is as follows:

| ID | Sex | Diagnosis | Age  [years] | Hoehn-Yahr stage | Years since diag. |
| --- | --- | --- | --- | --- | --- |
| 001 | M | PD | 67 | 2 | 4.5 |
| 002 | F | PD | 57 | 1 | 1 |
| 003 | F | PD | 62 | 1 | 3 |
| 004 | F | PD | 71 | 2.5 | 7 |
| 005 | F | PD | 68 | 2 | 1 |
| 006 | M | PD | 67 | 1.5 | 2 |
| 007 | F | PD | 73 | 1.5 | 2 |
| 008 | F | PD | 66 | 1.5 | 1 |
| 009 | F | PD | 76 | 2.5 | 3 |
| 010 | F | PD | 71 | 2.5 | 6 |
| 011 | F | PD | 59 | 1 | 2 |
| 012 | M | PD | 61 | 1 | 1 |
| 013 | M | PD | 71 | 2 | 5 |
| 014 | M | PD | 75 | 1.5 | 6 |
| 015 | F | PD | 72 | 1.5 | 2 |
| 016 | M | PD | 66 | 2 | 7 |
| 017 | M | PD | 64 | 2 | 5.5 |
| 018 | F | PD | 75 | 1.5 | 6 |
| 019 | F | PD | 61 | 1 | 2 |
| 020 | F | PD | 72 | 2 | 6 |
| 021 | F | PD | 72 | 2.5 | 5 |
| 022 | M | PD | 71 | 1 | 5 |
| 023 | F | PD | 73 | 1 | 1 |
| 024 | F | PD | 76 | 2 | 5 |
| 025 | F | PD | 56 | 1.5 | 4 |
| 026 | F | PD | 75 | 1.5 | 3 |
| 027 | M | PD | 68 | 2 | 2 |
| 028 | M | PD | 71 | 1.5 | 2 |
| 029 | M | HC | 73 | NA | NA |
| 030 | M | HC | 67 | NA | NA |
| 031 | M | HC | 64 | NA | NA |
| 032 | M | HC | 65 | NA | NA |
| 033 | F | HC | 73 | NA | NA |
| 034 | F | HC | 70 | NA | NA |
| 035 | F | HC | 62 | NA | NA |
| 036 | F | HC | 61 | NA | NA |
| 037 | M | HC | 72 | NA | NA |
| 038 | F | HC | 72 | NA | NA |
| 039 | F | HC | 77 | NA | NA |
| 040 | M | HC | 71 | NA | NA |
| 041 | F | HC | 71 | NA | NA |
| 042 | F | HC | 64 | NA | NA |
| 043 | F | HC | 75 | NA | NA |
| 044 | F | HC | 71 | NA | NA |
| 045 | F | HC | 61 | NA | NA |
| 046 | M | HC | 65 | NA | NA |
| 047 | M | HC | 61 | NA | NA |
| 048 | F | HC | 70 | NA | NA |
| 049 | M | HC | 70 | NA | NA |
| 050 | M | HC | 70 | NA | NA |
| 051 | F | HC | 67 | NA | NA |
| 052 | F | HC | 66 | NA | NA |
| 053 | M | HC | 67 | NA | NA |
| 054 | F | HC | 61 | NA | NA |
| 055 | M | HC | 65 | NA | NA |
| 056 | F | HC | 66 | NA | NA |
| 057 | F | HC | 74 | NA | NA |
| 058 | F | HC | 70 | NA | NA |
